# Prevention of Postpartum Depression by Multidisciplinary Collaboration in Japan

**DOI:** 10.31662/jmaj.2024-0070

**Published:** 2024-08-09

**Authors:** Shunji Suzuki

**Affiliations:** 1 Department of Obstetrics and Gynecology, Japanese Red Cross Katsushika Maternity Hospital, Tokyo, Japan

**Keywords:** mental health care, multidisciplinary collaboration, pregnant women, obstetric institute, Japan

## Abstract

This is an outline of the prevention of postpartum depression in obstetric institutes, with a focus on support through multidisciplinary collaboration in Japan. The onset of postpartum depression among women can be prevented by finding solutions to background factors causing mental health problems and providing multidisciplinary support.

## Introduction

Among women’s life cycles, pregnancy, childbirth, and child-rearing are extremely stressful periods due to the rapid and substantial changes in both their physical and emotional conditions, and they are also periods when mental disorders tend to develop, worsen, and flare up ^(1), (2)^. The ultimate outcomes of mental disorders are self-harm and other forms of harm; however, pregnancy, childbirth, and child-rearing were originally considered to be happy moments for women, and in the past, the suicide rate during pregnancy was thought to be lower than that among nonpregnant women of the same age ^(3)^. During pregnancy, it had been thought that the suicide rate would not increase even if a woman suffered from a mental disorder due to the various types of support available to pregnant women ^(3)^.

In Japan, postpartum depression occurs in 10%-15% of postpartum women ^(1)^. One of the main causes of postpartum depression in this country has been reported to involve social factors, such as an unwanted and/or unexpected pregnancy, lack of support, and unstable family situation ^(1)^. It has been estimated that about 50%-80% of women with postpartum depression exhibit relevant social factors since pregnancy. These social factors can increase the emotional burden of pregnancy and child-rearing. As it is impossible for medical staff alone to provide support for such social factors, support from multiple professions, including local administrative agencies, will be required. Meanwhile, early detection of psychological problems is impossible without a proactive approach by medical staff of obstetric institutions during pregnancy. Thus, cooperation from obstetric staff to multiple professionals will be needed.

In recent years, the proportion of pregnant women in Japan requiring support during pregnancy, including mental health care, has increased due to the shift to nuclear families, weakening of local communities, and increases in the numbers of older pregnancies, poor families, and unexpected pregnancies. In addition, if support is inadequate during the childbearing period, the mother may not be able to adapt to the new lifestyle of child-rearing, which results in the deterioration of her mental condition and the onset of postpartum depression ^(4)^.

Based on these background factors, we reviewed the prevention of perinatal mental disorders such as postpartum depression in obstetric institutes, with a focus on support through multidisciplinary cooperation.

## Prevention of Postpartum Depression by Multidisciplinary Collaboration as a Population Approach

The ultimate goal of measures for maternal mental disorders is to support a healthy child-rearing environment through the provision of appropriate support for the unstable and vulnerable changes in the mother’s mind. It is also known that multidisciplinary support through a high risk approach, including local administration for breastfeeding and daily living in the field of obstetrics, is effective for mild cases, which account for a large proportion of these cases ^(5), (6), (7), (8)^. As the possibility of relapse or serious illness cannot be ruled out at any time, the reassurance of the cooperation and availability of psychiatric departments, particularly in emergent cases, is a backstop ^(6), (7), (8), (9), (10)^.

Cases requiring childcare support related to postpartum depression, etc., include those in which the childcare environment is considered to be inadequate before delivery, psychiatric symptoms such as depression are observed, and inappropriate childcare attitudes and behaviors are feared due to negative feelings toward the fetus/newborn ^(1)^. Although perinatal to postpartum depression can occur in 10%-15% of cases ^(1)^, it should be noted that mothers with depression tend to not seek help on their own ^(1), (11), (12)^. However, the timing of onset is characteristic, such as early pregnancy and early postpartum, and risk factors such as unexpected pregnancy, strong anxiety regarding pregnancy, history of mental disorder, lack of support, and unstable family situation have been identified to some extent ^(1)^. Moreover, more than half of women who develop postpartum depression exhibit depressive symptoms during pregnancy, and there is evidence proving that intervention and support during pregnancy can prevent the onset and severity of postpartum depression by identifying the high risk groups ^(1), (11), (12)^. Furthermore, pregnant and postpartum women, with some exceptions, need to attend regular checkups even if they have no symptoms. Based on the above, we believe that the key points are 1) to recognize the need for a proactive approach from the medical side, 2) to conduct a population approach during the first trimester of pregnancy and at 1-2 months postpartum when signs of a deteriorating mental status are likely to be recognized, and 3) to provide regular intervention as preventive support for the screened high risk group ^(1), (7)^. The perinatal period is a time of regular health checkups for pregnant women, including the healthy ones, and a time when medical personnel have regular contact with them. In particular, nursing professionals, such as midwives, nurses, and public health nurses, are in a position to identify changes in the mental state and family relationships of pregnant women and provide continuous information and intervention during antenatal and maternity checkups ^(1), (7)^. Thus, obstetricians and nursing professionals need to acquire communication skills in preparation for providing support; in recent years, several related organizations, including the Japan Obstetricians and Gynecologists Association (https://www.jaog.or.jp/), have been holding regular training sessions on how to communicate with pregnant and postpartum women using questionnaires ^(1)^.

## Prevention of Postpartum Depression by Multidisciplinary Collaboration as a High Risk Approach

In obstetric medical institutions in Japan, depending on the size of the institution, support is provided in collaboration with clinical psychologists, medical social workers, medical staff, and others ^(5), (6), (7)^. In some general hospitals, a psychiatric nurse specialist certified by the Japan Nursing Association (https://www.nurse.or.jp/) provides preventive and early intervention for mental disorders, psychological intervention such as psychoeducation and cognitive behavioral therapy, and support for decision-making regarding psychotropic medication, monitors the mental status of the patients, and enables interdisciplinary coordination, e.g., consultation with support providers ^(1), (4)^. This suggests that preventing the onset of postpartum depression among women is possible by finding solutions to background factors causing their mental health problems and providing multidisciplinary support.

Maternal mental disorders, except for postpartum depression, have been demonstrated to have a low incidence but require coordinated pharmacotherapy with psychiatry in most cases as inadequate treatment is associated with a high rate of severe illness and suicide ^(1), (13)^. Meanwhile, perinatal and postpartum depression can occur in 10%-15% of women without adequate support, and once it worsens, medication is necessary. This may be a reason why almost all Japanese obstetricians often think that “mental disorder = postpartum depression” ^(1), (13)^.

In 2010, “specified expectant mothers (SEMs)” were defined by the Japanese Ministry of Health, Labor and Welfare as pregnant women at a high risk of abuse and/or in need of extra support after birth due to some social problems, such as unstable income and mental disorders. For SEMs needing psychological support, the cooperation of their partners and families is important; during the childbearing and childcare periods, they also need continuous support from their families and the local government ^(7), (13)^. Therefore, it is preferable for them to give birth as close to their residence as possible rather than being referred to a distant tertiary hospital ^(13)^. Thus, unless the case is serious enough to pose a risk of self-injury or harm to others, we should focus on creating a supportive environment that facilitates connection to the local government in the area where the child will reside. Meanwhile, Japan has a custom of “homecoming delivery,” and in some areas, most SEMs give birth outside their place of residence ^(1), (14)^. The Japanese homecoming delivery called “satogaeri bunben” has been defined as follows: a pregnant woman returns to her family home for delivery at 32-34 weeks of gestation and stays with her parents and family members to get sufficient support and to physically and psychologically rest until a couple of months after delivery. If the mental state of pregnant and postpartum women remains healthy, the homecoming delivery is a good opportunity to learn child-rearing in Japan, where the nuclear family has become increasingly common. It could also prevent childcare anxiety ^(14)^. Experience, skill, and wisdom about care of the baby can be passed on from the grandmother to the new mother. If the SEM has a good relationship with her biological mother, receiving advice on childcare while resting at the family home until the mother’s sufficient physical recovery may also help prevent the onset of postpartum depression. However, there are cases in which the mother’s mental condition deteriorates after returning to her place of residence when support is no longer provided. In such cases, it is important to note that the rate of sudden visits to obstetricians and psychiatrists is high, and it is important to create an environment in which it is easy to see the original doctor if something goes wrong after returning home and to establish cooperation among local governments ^(13), (14), (15)^.

## Conclusion

In conclusion, we have outlined the prevention of postpartum depression in obstetric institutes, with a focus on support through multidisciplinary cooperation.

## Article Information

### Conflicts of Interest

None

### Author Contributions

Shunji Suzuki: all project development, management, analysis, and manuscript writing/editing.

### Consent

The study protocol was approved by the Ethics Committee of the Japanese Red Cross Katsushika Maternity Hospital (K2023-11).
